# Muscle stem cell adaptations to cellular and environmental stress

**DOI:** 10.1186/s13395-022-00289-6

**Published:** 2022-02-12

**Authors:** Maria Vittoria Gugliuzza, Colin Crist

**Affiliations:** 1 Department of Human Genetics, McGill University, 3640 University St, Montréal, H3A 0C7 Canada; 2 Lady Davis Institute for Medical Research, Jewish General Hospital, 3755 chemin de la Côte Ste. Catherine, Montréal, H3T 1E2 Canada

**Keywords:** Muscle stem cell, MuSC, Stress response pathways, Translational control of gene expression

## Abstract

**Background:**

Lifelong regeneration of the skeletal muscle is dependent on a rare population of resident skeletal muscle stem cells, also named ‘satellite cells’ for their anatomical position on the outside of the myofibre and underneath the basal lamina. Muscle stem cells maintain prolonged quiescence, but activate the myogenic programme and the cell cycle in response to injury to expand a population of myogenic progenitors required to regenerate muscle. The skeletal muscle does not regenerate in the absence of muscle stem cells.

**Main body:**

The notion that lifelong regeneration of the muscle is dependent on a rare, non-redundant population of stem cells seems contradictory to accumulating evidence that muscle stem cells have activated multiple stress response pathways. For example, muscle stem cell quiescence is mediated in part by the eIF2α arm of the integrated stress response and by negative regulators of mTORC1, two translational control pathways that downregulate protein synthesis in response to stress. Muscle stem cells also activate pathways to protect against DNA damage, heat shock, and environmental stress. Here, we review accumulating evidence that muscle stem cells encounter stress during their prolonged quiescence and their activation. While stress response pathways are classically described to be bimodal whereby a threshold dictates cell survival versus cell death responses to stress, we review evidence that muscle stem cells additionally respond to stress by spontaneous activation and fusion to myofibres.

**Conclusion:**

We propose a cellular stress test model whereby the prolonged state of quiescence and the microenvironment serve as selective pressures to maintain muscle stem cell fitness, to safeguard the lifelong regeneration of the muscle. Fit muscle stem cells that maintain robust stress responses are permitted to maintain the muscle stem cell pool. Unfit muscle stem cells are depleted from the pool first by spontaneous activation, or in the case of severe stress, by activating cell death or senescence pathways.

## Background

**Fig. 1:**
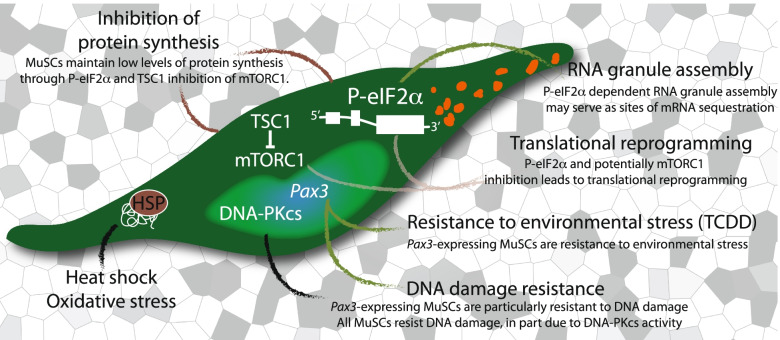
Living outside the comfort zone: quiescent MuSCs adapt to cellular and environmental stress. A stylised MuSC (green) highlighted over the skeletal muscle myofibres (grey). MuSCs maintain low levels of protein synthesis by phosphorylation of eIF2α and TSC1 inhibition of mTORC1 activity. Genetic inactivation of these stress response pathways in MuSCs leads to G_alert_ or spontaneous activation. P-eIF2α leads to the assembly of DDX6(+) RNA granules (orange) in MuSCs, which resemble stress granules and may functionally serve as sights of mRNA sequestration. P-eIF2α also leads to translational reprogramming of mRNAs that confer stem cell properties on quiescent, self-renewing, or expanding MuSCs. In the nucleus of quiescent MuSCs (light green), elevated DNA-PKcs levels ensure efficient and accurate DNA repair. Expression of the paired homeodomain transcription factor *Pax3* marks a subset of MuSCs with increased resistance to multiple stresses, including DNA damage and environmental pollutants. MuSCs that do not express *Pax3* spontaneously activate when challenged with the environmental pollutant TCDD. Quiescent MuSCs express mRNAs for heat shock proteins *Hsp40*, *Hsp70*, and *Hsp90* (brown) and upregulate these chaperones during early activation. These chaperones may be required to counteract the accumulation of misfolded proteins

**Fig. 2:**
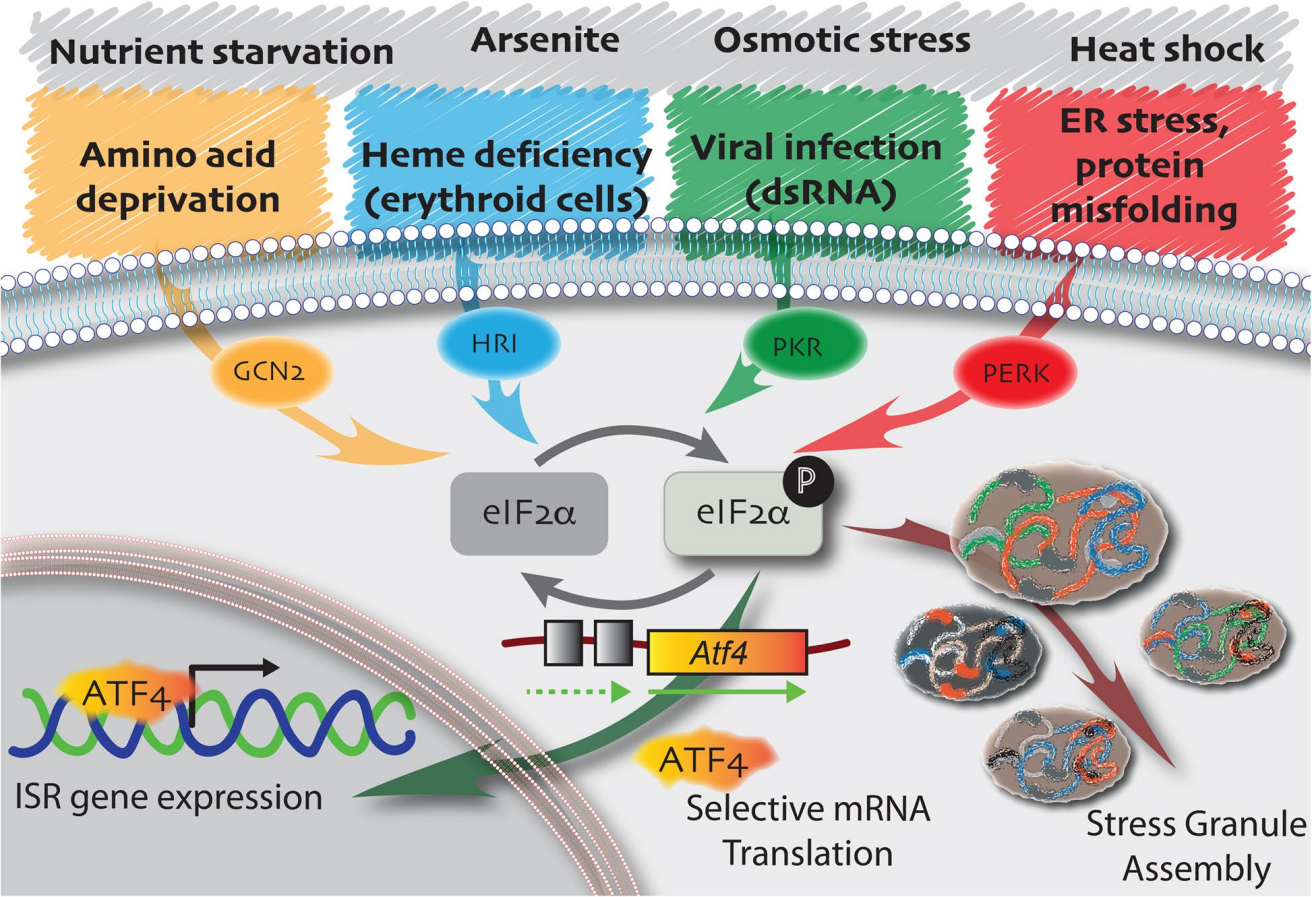
The integrated stress response. In response to various sources of cellular and environmental stress, a family of four eIF2α kinases phosphorylate (black P) eIF2α (light grey). GCN2 (yellow) responds to amino acid deficiency, HRI (blue) responds to heme deficiency in erythroid cells, and PKR (green) responds to the presence of double-stranded RNA coincident with viral infection and PERK (red) responds to endoplasmic reticulum stress. Additional stresses that phosphorylate eIF2α, for which the corresponding kinase is unknown, are indicated (grey). Phosphorylated eIF2α leads to a global repression of translation. Accumulation of a pool of mRNAs, stalled at the initiation step of translation, seed the assembly of stress granules (maroon arrow). In contrast P-eIF2α reprograms translation to favour a subset of mRNAs, such as those for activating transcription factor *Atf4* (orange), that contain uORFs in their 5’UTR. P-eIF2α-dependent translation of *Atf4* and subsequent nuclear localisation of ATF4 protein initiate the integrated stress response (dark green arrow), a pro-survival pathway

The skeletal muscle efficiently regenerates after acute injury in part due to a population of resident adult muscle stem cells (MuSCs), also named ‘satellite cells’ for their anatomical position sandwiched between the myofibre and the basal lamina [1]. Normally mitotically quiescent (G_0_ phase), MuSCs express members of the paired box (*Pax*) family of transcription factors *Pax7*, and in a subset of muscle, *Pax3*. MuSCs are primed to rapidly enter the myogenic programme, in part because they accumulate transcripts for myogenic regulatory factors myogenic factor 5 (*Myf5)* and myogenic determination protein (*MyoD*), along with cell cycle genes like *Dek*, which remain repressed by the action of microRNA and ribonucleic acid (RNA) binding proteins [2–6]. Some accumulating messenger RNA (mRNA), like those for *Myf5*, are translated inefficiently and further sequestered in cytoplasmic RNA granules [2] (Fig. 1). The formation of RNA granules in quiescent MuSCs requires the phosphorylation of eukaryotic initiation factor 2α (P-eIF2α) [7] (Fig. 1), a component of the integrated stress response (ISR) [8] (Fig. 2). In response to injury, the earliest stages of MuSC activation include the rapid dissociation of RNA granules and the accumulation of MYF5 and MYOD protein [2, 7, 9, 10]. Within hours after injury but prior to the first cell division, activated MuSCs also initiate a new transcriptional response to stress, rapidly upregulating core stress genes in ontologies for the stress activated p38-mitogen-activated protein kinase (p38MAPK) and transcription in response to stress [11]. Activated MuSCs re-enter the growth 1 (G_1_) phase of the cell cycle, proliferate extensively to expand the population of myogenic progenitors that are required to efficiently regenerate muscle, and self-renew to replace the endogenous MuSC pool for future rounds of regeneration.

The maintenance of the MuSC pool is critical for lifelong regeneration of the skeletal muscle, and yet, MuSCs are under pressure by, and have adapted to, numerous sources of cellular stress (Fig. 1). Not unlike yeast and all microorganisms, MuSCs spend most of their lifetime in a prolonged state of quiescence, which is also an evolutionary conserved response of cells that encounter environmental stress, poor nutrient availability, limited oxygen, and poor sources of cellular energy [12, 13]. The skeletal muscle is considered a hypoxic environment, and in particular, the MuSC niche sandwiched between the myofibre and basal lamina is hypoxic [14]. Although the majority of MuSCs reside in close proximity to the blood vessels, up to 15% of MuSCs reside away from the vessels where hypoxia may be even greater [15]. Quiescent MuSCs also have poor sources of cellular energy. They have few mitochondria and generate low amounts of cellular adenosine triphosphate (ATP) compared to activated MuSCs [16]. Quiescent MuSCs exhibit low metabolism, which may preserve MuSC regenerative potential by limiting the production of reactive oxygen species (ROS). Low metabolism is dependent on fatty acid oxidation and oxidative phosphorylation, which in turn promotes epigenetic modifications that silence the myogenic programme [17].

Upon acute injury, MuSCs break quiescence and reactivate the cell cycle. Activation is associated with a metabolic shift to anaerobic glycolysis, which is needed to support the increased cellular demands of cell growth and proliferation [17, 18]. Activated MuSCs potentially encounter proliferative stress during their critical expansion phase needed to rapidly generate a population of myogenic progenitors required for muscle regeneration. The proliferative stress encountered by MuSCs in the normal regeneration of the muscle is illustrated by the accelerated decline in proliferation potential exhibited by myoblasts isolated from the skeletal muscle of Duchenne muscular dystrophy (DMD) patients [19]. Moreover, the critical expansion phase of MuSCs responding to muscle injury takes place within a regenerative environment characterised by pro-inflammatory cytokines with potential for cytotoxicity [20] and increased oxidative stress [21].

In this review, we highlight the consequences of a decline in MuSC fitness that is evident in muscle disease and summarise mechanisms of stress resistance and adaptation that MuSCs use to maintain the fidelity of the MuSC pool. We provide examples by which MuSCs not only survive, but possibly thrive under stressful conditions to regenerate the skeletal muscle. We propose that MuSCs have not only adapted to stress, but also thrive under stressful conditions, using basal levels of cellular and environmental stress to ensure their fitness.

## Main text

### Maintenance of the MuSC pool is essential for regeneration of the skeletal muscle

MuSCs are a rare population of cells in the skeletal muscle, making up less than 5% of all nuclei in the skeletal muscle. The importance of the integrity of the MuSC pool was elegantly illustrated by genetic approaches, whereby the *Pax7*-expressing MuSC pool was conditionally ablated by the expression of diphtheria toxin (DTA) after tamoxifen administration to *Pax7*^*CreERT2/*+^; *R26*^*DTA*^ mice, with consequent collapse of the skeletal muscle regeneration after acute injury [22–24]. These studies demonstrate that the skeletal muscle does not regenerate without MuSCs and also confirm that MuSCs are the only cell population with myogenic potential that regenerates muscle in vivo [25]. With only a single and rare cellular source fueling lifelong regeneration of the skeletal muscle, it is critical that this population is maintained.

### A decline in skeletal muscle regeneration occurs when the integrity of the MuSC pool is compromised

The importance of maintaining the MuSC pool is also illustrated in muscle disease, the progression of which often coincides with a loss of MuSC numbers and function. DMD is a devastating X-linked skeletal muscle degenerative disease affecting approximately 1 in 5000 boys [26] with 100% mortality by early adulthood. The disease is caused by mutations in the *DMD* gene, which lead to impaired synthesis of full-length dystrophin protein, the absence of which causes myofibre fragility. In DMD patients, cycles of muscle degeneration and regeneration lead to the exhaustion of the MuSC pool, in part because chronically activated MuSCs exhibit severe proliferation defects and undergo premature senescence [19]. Dystrophin protein is now understood to be expressed in activated MuSCs, where it regulates MuSC polarity and asymmetric cell divisions that are required to maintain the MuSC pool [27, 28]. In the *Dmd*^*mdx*^ mouse model of DMD, dystrophin deficiency also leads to chronic degeneration of skeletal muscle. However, the phenotype is mild and *Dmd*^*mdx*^ mice have a normal lifespan, in part due to greater proliferation capacity of mouse MuSCs that fuels regeneration of the muscle [29] and likely in part due to their shorter (27 months) lifespan. In addition to DMD, mutations in *PAX7* have also been linked to the pathology of a new myopathy with variable severity in humans. The lack of *PAX7*-expressing MuSCs in the human muscle may lead in part to muscle atrophy, hypotonia, scoliosis, and mild dysmorphic facial features that are present in individuals with these mutations [30].

The progression of the muscle wasting associated with sarcopenia is also accompanied by a decrease in the fitness and numbers of MuSCs [31], with consequent loss of skeletal muscle regeneration [32, 33]. Compared to MuSCs isolated from young adult muscle, MuSCs isolated from old mice are prone to apoptosis and senescence when placed in culture [32, 34]. Aged MuSCs appear to activate a number of stress response pathways associated with p38^MAPK^ [34–36], which is activated in response to a variety of cell stress and inflammation [37], Jak-Stat3 [38, 39], a pro-survival pathway activated in response to stress [40] and p16^INK4a^ [32], which negatively regulates the cell cycle in response to cell stress. In young adult MuSCs, *p16*^*INK4A*^ expression is epigenetically silenced. In geriatric mice, ubiquitination of H2A leads to permissive chromatin marks that enable *p16*^*INK4A*^ expression. MuSCs with elevated *p16*^*INK4A*^ expression do not activate and transit into the G1 phase, but instead irreversibly become senescent [32], with consequent depletion of the functional MuSC pool and impaired muscle regeneration.

Altogether, genetic ablation strategies and myopathies that are characterised by a loss of MuSCs number and function illustrate the importance of maintaining the MuSC pool to fuel lifelong regeneration of the muscle. The notion that the tissue microenvironment, or niche, protects MuSCs from cellular and environmental stress is challenged by evidence that quiescent MuSCs actively initiate multiple stress response pathways (Fig. 1). Next, we review the cellular responses to stress utilised by MuSCs and further discuss the fate of MuSCs when these stress response pathways are compromised.

### MuSC adaptations to cellular stress

#### MuSC responses to stress by reprogramming mRNA translation

Regulation of mRNA translation contributes to many aspects of cell physiology, including cell growth, proliferation, differentiation, and cell survival when exposed to stress. The coordinated regulation of transcription and translation provides optimal levels of required proteins that is balanced against the energy expenditure of protein synthesis [41, 42]. Under conditions of stress, the survival of cells depends on the rapid reprogramming of translation to selectively translate mRNAs required to initiate a stress response, while globally repressing mRNA translation to reduce the energy requirements of protein synthesis [43, 44].

**Fig. 3:**
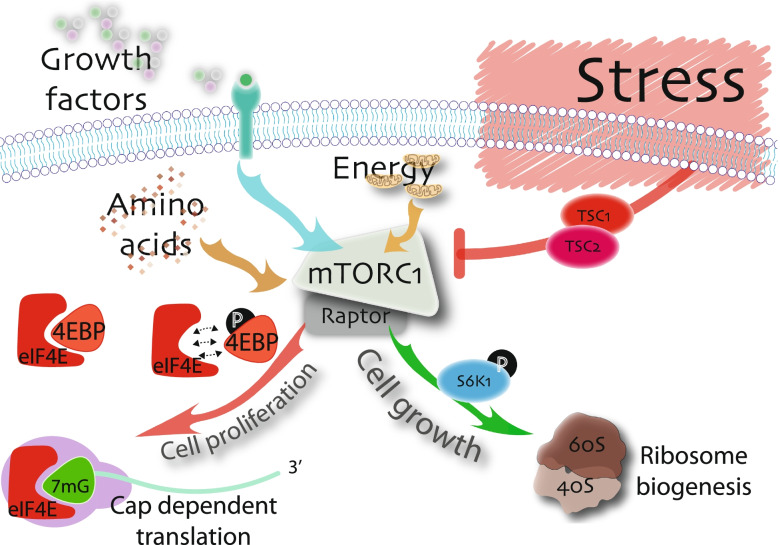
The mTORC1 pathway in stress. The presence of growth factors (grey, green, purple circles), abundant amino acids (brown diamonds), and cellular energy (light brown mitochondria) positively regulated mTORC1 to regulate cell growth pathways through the phosphorylation of S6K1 kinase (green arrow), and cell proliferation pathways (red arrow) through the phosphorylation of eIF4E binding protein (4E-BP). Phosphorylated 4E-BP no longer competes for eIF4E binding, permitted eIF4E to initiate cap-dependent translation. eIF4E is the cap binding protein that functions within the eIF4F tertiary complex (purple) along with eIF4G and eIF4A (not shown). Cellular and environmental stress activates the TSC1/TSC2 complex to inhibit mTORC1 signalling, leading to the repression of these pathways, with consequent decrease in cell growth and proliferation

The arrest of translation initiation, the rate limiting step of protein synthesis, is a major hallmark of stress-induced translational control. Two translation initiation factors play central roles in the regulation of mRNA translation in response to stress. These are eukaryotic initiation factor 2 (eIF2), which is central to the ISR [45] (Fig. 2) and eukaryotic initiation factor 4E (eIF4E), which is a key component of a stress response regulated by the mechanistic target of rapamycin complex 1 (mTORC1) signalling pathway (Fig. 3) via eIF4E binding proteins (4E-BPs) [46].

#### The integrated stress response

In response to a broad range of cellular stress, eukaryotes activate the ISR [8, 45] (Fig. 2). The central event in this pathway is the phosphorylation of eukaryotic initiation factor 2α (P-eIF2α) by one of four members of the eIF2α kinase family. General control nonderepressible 2 (GCN2) responds to amino acid starvation [8], protein kinase R (PKR) responds to the presence of viral double-stranded RNA [47], heme-regulated inhibitor (HRI) responds to the absence of heme in erythroid cells [48, 49], and PKR-like endoplasmic reticulum kinase (PERK) is activated in response to endoplasmic reticulum stress [50]. Additional environmental stresses that induce eIF2α phosphorylation for which the specific kinase remains unknown are exposure to arsenite, osmotic stress, heat shock, and nutrient starvation (Fig. 2).

The eIF2 complex (eIF2α, eIF2β, and eIF2γ) is a trimeric protein complex that is essential for protein synthesis and responsible for recycling the methionine loaded tRNA (Met-tRNA) initiation complex to the 40S ribosomal subunit to form the 43S preinitiation complex. P-eIF2α turns eIF2 into a competitive inhibitor of the guanine nucleotide exchange factor eIF2B, to prevent recycling of the eIF2-GTP-initiatior methionyl tRNA ternary complex needed to initiate translation [51]. The resultant block in translation initiation has two important consequences to initiate a stress response (Fig. 2). First, translation reprogramming occurs in the cell whereby a global arrest in translation of mRNA is countered by selective translation of specific mRNAs required for the initiation of a stress response. Selective mRNA translation is mediated in part by inhibitory upstream ORFs (uORFs) in the 5’UTRs of transcripts, exemplified by transcripts for activating transcription factor 4 (*Atf4*) [52] (Fig. 2). P-eIF2α-dependent readthrough of inhibitory uORFs in the 5’UTR of *Atf4* enables the initiation of translation at the main ORF encoding for ATF4, and ATF4 in turn activates the expression of genes required for cell recovery in response to stress [53]. Although the ISR is a pro-survival pathway, exposure to severe stress or prolonged stress leads to the induction of cell death pathways [54–56]. Cells that have genetic modifications to remove the phosphorylated serine residue at position 51 of eIF2α (S51A) are unable to cope with acute stress. Moreover, the importance of eIF2α phosphorylation in mammals is illustrated by perinatal lethality in *eIF2α*^*S51A/S51A*^ mice [57].

Second, P-eIF2α leads to a pool of mRNAs paused at the initiation step of translation, which through liquid-liquid phase separation seed the assembly of stress granules, membrane-less organelles of ribonucleoprotein complexes composed of RNA binding proteins and stalled mRNAs [48, 49] (Fig. 2). When the eIF2-GTP-initiatior methionyl tRNA ternary complexes are reduced, RNA binding proteins TIA1 and TIAR promote the assembly of non-canonical preinitiation complexes that lack the methionine loaded tRNA. TIA1 and TIAR dynamically triage translationally incompetent mRNAs into stress granules [58]. Despite that stress granule composition, assembly and disassembly have been studied for many years, their true function in the cell remains unclear. They presumably serve as sites of mRNA triage, help the cell cope with stress, and possibly facilitate the recovery and rapid reinitiation of translation after stress removal and stress granule disassembly [43, 59].

#### The phosphorylation of eIF2α is a translational control mechanism regulating MuSC quiescence and self-renewal

Quiescent MuSCs maintain low levels of protein synthesis, by PERK phosphorylation of eIF2α. The activity of PERK and P-eIF2α are both essential for MuSC quiescence and self-renewal [7]. Upon MuSC activation, eIF2α is rapidly dephosphorylated, coincident with translation and rapid accumulation of myogenic regulatory factors MYF5 and MYOD. When cultured ex vivo, rare MuSCs expressing only PAX7 maintain P-eIF2α, while the bulk of proliferating MuSCs that activate the myogenic programme dephosphorylate eIF2α. Like all cells, MuSCs require P-eIF2α to initiate a pro-survival stress response when challenged with an acute stress, for example brief exposure to ER stress inducer thapsigargin. However, MuSCs do not require P-eIF2α for cell survival under physiological conditions, nor is P-eIF2α required for MuSC survival during a regenerative response after acute injury. Instead, MuSCs that are unable to phosphorylate eIF2α are prone to spurious activation, proliferation, and contribution to new or existing myofibres in vivo [7].

P-eIF2α is also required for the assembly of RNA granules within the cytoplasm of quiescent MuSCs [7] (Fig. 1). These RNA granules are similar to size and RNA binding protein composition to stress granules, marked by RNA binding proteins DDX6, TIAR, FMRP, and GW182 [2, 3, 6]. They do not contain mRNA decapping enzyme DCP1, which is a marker of P bodies that are considered sites of mRNA decay. Instead, DCP1-positive P-bodies predominate in activated MuSCs [6]. Quiescent MuSC RNA granules are thought to be sites of storage for transcripts required for activation of the myogenic programme and proliferation. For example, *Myf5* transcripts colocalize to RNA granules and can be immunoprecipitated with antibodies against DDX6 [2]. Upon MuSC activation, the dissolution of RNA granules and rapid accumulation of MYF5 protein are amongst the earliest markers of MuSC activation, which coincides with reengagement of *Myf5* mRNA with translating ribosomes and rapid accumulation of MYF5 protein. Therefore, RNA granules possibly ‘prime’ quiescent MuSCs for rapid activation by their disassembly and rapid initiation of *Myf5* mRNA translation [2, 7].

MuSCs appear not only to activate the P-eIF2α stress response pathway to maintain quiescence and self-renewal, but also thrive under ex vivo conditions that promote eIF2α phosphorylation [7, 60]. Under normal culture conditions, a subset of PAX7-expressing MuSCs maintain P-eIF2α, while activated MuSCs that express MYOD dephosphorylate eIF2α. Fresh isolated MuSCs that are cultured in the presence of the eIF2α phosphatase inhibitor sal003 expand ex vivo as a population of PAX7(+), MYOD(-) cells. These cells retain their stem cell properties to regenerate muscle and self-renew, illustrated by their engraftment into the *Dmd*^*mdx*^ preclinical mouse model of Duchenne muscular dystrophy [7].

How MuSCs expand under culture conditions that promote the eIF2α phosphorylation stress response and lower global rates of protein synthesis is an interesting paradox that is potentially resolved by translational reprogramming. Culture of MuSCs in the presence of sal003 revealed hundreds of genes that are upregulated at the level of protein, without a corresponding increase in mRNA levels [61], suggesting post-transcriptional regulation. The most significantly represented class of genes were for those involved in spindle assembly, suggesting that *Pax7*-expressing MuSCs use eIF2α phosphorylation to maintain the fidelity of cell division. For example, P-eIF2α enables the translation of an mRNA for the mitotic spindle assembly gene transforming acidic coiled coil protein 3 (*Tacc3*) by virtue of inhibitory uORFs present in the 5’ untranslated region (5’UTR) of *Tacc3* mRNA. In the absence of *Tacc3*, MuSCs expand poorly, with consequent depletion of the integrity of the MuSC pool and compromised regeneration of the skeletal muscle after acute injury [61].

There are a number of remaining questions related to the eIF2α pathway in MuSCs. What are the identity and fate of mRNAs that localise to P-eIF2α dependent RNA granules? Conversely, which mRNAs are translated in a P-eIF2α-dependent manner in quiescent MuSCs? These questions are potentially addressed with next generation RNA-seq technologies compatible with low amounts of mRNA isolated from ribosomes [60] or new strategies to isolate and determine the RNA component of RNA granules [61, 62]. Another important question is to what extent is P-eIF2α dependent changes in mRNA translation modified in muscle disease. While in normal healthy muscle, MuSC quiescence is maintained by PERK phosphorylation of eIF2α, the extent to which eIF2α phosphorylation is modified by kinases responding to other forms of cellular stress, for example PKR or GCN2, within the context of aging or chronically degenerating muscle, remains unclear.

#### MuSC quiescence is mediated in part by the stress response pathway regulated by mTORC1

Mechanistic target of rapamycin (mTOR) is a serine threonine kinase belonging to the family of phosphatidylinositol 3-kinase (PI3K)–related kinase (PIKKs) and is a main activator of the cellular biosynthesis machinery needed for increase cell growth and proliferation [62]. Mechanistically, mTOR functions in multiprotein complexes mTORC1 (Fig. 3) and mTORC2 and is activated by growth factors, nutrients, and energy [63]. The two most extensively studied downstream effectors of mTORC1 signalling are p70 S6 kinase (p70^S6K^; RPS6K1/2) and the eIF4E binding protein 1/2/3 family (4E-BP) (Fig. 3). p70^S6K^ regulates cell growth by phosphorylation of ribosome protein S6 to increase rates of ribosome biogenesis and protein synthesis [63, 64] (Fig. 3). Phosphorylation of 4E-BPs regulates cell proliferation by disrupting their inhibition of eIF4E to enable 7-methylguanosine 5-triphosphate (m7GTP) cap-dependent translation of mRNAs encoding cell cycle regulators [46] (Fig. 3). Translational reprogramming of mRNA is also a feature of the mTORC1 pathway, since 4E-BPs regulate the translation of specific mRNAs that have established 5’ terminal oligopyrmidine (TOP) motifs [65].

The activity of mTOR is sensitive to complex signalling networks, including those that are activated in response to cell stress. The bulk of mTORC1 inhibition is channelled through the tuberous sclerosis (TSC) proteins TSC1 and TSC2 [62] (Fig. 3), which together serve to promote inactivating GTP hydrolysis of components of mTORC1. Cellular stresses that activate TSC1/TSC2 include growth factor deficiencies, low cellular energy, hypoxia, ROS, and DNA damage [62]. The resultant decrease in p70^S6K^ activity leads to decreased ribosome biogenesis and reduced cell growth. The decrease in phosphorylated 4E-BP enables 4E-BP binding to eIF4E, leading to inhibition of cap dependent mRNA translation, and reduced cell proliferation (Fig. 3).

#### Quiescent G_0_ MuSCs activate mTORC1 signalling to transition to G_alert_

An important role for mTORC1 signalling has been elucidated in the early activation of MuSCs, termed G_alert_ [16]. Tissue injury at distal sites leads to the accumulation and circulation of growth factors like hepatocyte growth factor (HGF) that activate mTORC1 to increase rates of protein synthesis [66]. The G_alert_ phase is characterised by increased mitochondria, more ATP and an increase in cell size, but not by an increase in cell proliferation. Mechanistically, the G_alert_ phenotype, characterised by an increase in MuSC size, is associated with an increase in S6 kinase phosphorylation. Moreover, genetic inactivation of *Tsc1* specifically in *Pax7*-expressing MuSCs leads to acquisition of the G_alert_ phenotype independent of distal injury, suggesting that MuSC quiescence is also regulated by cellular or environmental stresses that together inhibit mTORC1 signalling via TSC1 (Fig. 1). Altogether, the inhibition of mTORC1 signalling by TSC1 maintains MuSC quiescence, while the activation of mTORC1 by circulating growth factors like HGF is an early stage of MuSC activation [16, 66].

How the cell growth arm of the mTOR pathway, regulated by S6 phosphorylation, is specifically activated in G_alert_, while the cell proliferation arm of the mTOR pathway, potentially regulated by 4E-BP, remains resistant, is unknown. Positive mTORC1 regulation of cell proliferation potentially becomes the dominant response in activated MuSCs, since inactivation of Raptor, a specific component of the mTORC1 signalling pathway, limits MuSC proliferation, with consequent perturbation in muscle differentiation and regeneration [67]. Lastly, mTORC2 is a second mTOR complex that responds to growth factors to regulate cell proliferation, but the study of mTORC2 has lagged behind mTORC1 and has also not yet been investigated within the context of MuSC quiescence and activation.

### The DNA damage response

When challenged with irradiation induced genotoxic stress, MuSCs resist apoptosis compared to non-myogenic cells and differentiated muscle present in the skeletal muscle. Mechanistically, quiescent MuSCs more accurately and efficiently repair DNA double-stranded breaks (DSBs) than activated MuSCs and committed progeny. Resistance to DNA damage is mediated in part due to the activity of DNA-PKcs [68] (Fig. 1), which is a central effector of the DNA damage response (DDR), a stress response pathway that senses DNA damage and replication stress to activate a protective response. DNA-PKcs is another member of the PIKK kinase family that function to phosphorylate a large number of substrates that are required for efficient and accurate DNA repair and also coordinate DNA repair with stalls on transcription, replication, and cell proliferation. While all quiescent MuSCs exhibit increased DNA damage repair compared to their activated and differentiated progeny, the subset of *Pax3*-expressing MuSCs is particularly resistant to genotoxic stress (Fig. 1). These cells have reduced levels of ROS, exhibit low levels gamma histone family member X (γH2Ax) foci, and reduced DNA damage in response to irradiation than MuSCs that only express *Pax7*. These cells are rare, exhibit limited contribution to normal regeneration and repair, but exhibit stress tolerance and are capable of clonal expansion and contribution to repair under stress [69].

### Environmental stress

Quiescent MuSCs have developed resistance to xenobiotics, genotoxics, and oxidative stress. Toxic substances may be pumped out of the quiescent MuSC by virtue of high-level expression of genes for efflux channels *Abcb1a*, *Abca5*, and *Abcc9*. Moreover, quiescent MuSCs may have developed strategies to solubilise toxic substances. The aryl hydrocarbon receptor (Ahr) is also expressed at high levels in MuSCs, where it plays a role to sense toxic molecules like dioxin derivatives or polycyclic aromatic hydrocarbons [70]. Of significant interest, exposure of mice to environmental stress by the injection of the environmental pollutant 2,3,7,8-tetrachlorodibenzo-p-dioxin (TCDD) leads directly to MuSC activation and fusion to myofibres in an AhR dependent manner, while only a minor fraction of MuSCs exhibit impaired survival. In contrast, the subset of *Pax3*-expressing MuSCs express low levels of AhR and resist TCDD exposure (Fig. 1). When adult mice are treated with TCDD for a period of 10 weeks, numbers of MuSCs expressing *Pax7* only decrease, while *Pax7*/*Pax3*-expressing MuSCs remain unchanged [71].

### The heat shock response

The heat shock response is a pro-survival pathway first described as a signalling response to elevations in temperature. However, many stresses activate the heat shock response, including the accumulation of protein aggregates caused by oxidative stress which in turn can be caused by exposure to heavy metals and pollutants [72–74]. To counteract the presence of protein aggregation, cells upregulate the expression of chaperone proteins that help fold nascent proteins correctly, refold misfolded proteins, and clear protein aggregation. Overall rates of transcription and mRNA translation are reduced to alleviate the burden of misfolded proteins, while protective genes are selectively expressed such as the heat shock factors (HSFs) HSF1, HSF2, and HSF4 [72]. HSFs translocate to the nucleus where they activate the expression of chaperones designated as the heat shock proteins (Hsp) such as *Hsp27* and *Hsp70*. These HSPs promote cell survival by inhibiting apoptosis pathways and by refolding proteins [72, 73, 75].

Transcripts for heat shock proteins in the HSP40, HSP70, and HSP90 family of chaperones increase in fresh isolated, early activated MuSCs, although these transcripts are also abundant in quiescent MuSCs in vivo [76] (Fig. 1). Although it remains unclear the extent to which HSPs are involved in MuSC quiescence and activation [77], a mild heat shock to donor derived myoblasts was sufficient to induce HSP70 expression, increase cell survival after exposure to an acute stress, and improve the engraftment of these myoblasts into the *Dmd*^*mdx*^ mouse model of DMD [78].

### Perspective: cellular stress tests maintain the fitness of the MuSC pool

Environmental and cellular stress cause the cell to activate pathways that allow the cell to cope with the stress and activate an appropriate protective response. Conversely, if the stress is too severe or prolonged, stress response pathways eventually lead to senescence or the activation of cell death pathways that lead to apoptosis, autophagic cell death, and necrosis [73, 79, 80]. These cell fate decisions, cell survival if stress is resolved versus cell death if stress is too severe, are cellular responses to a variety of stress including DNA damage, heat shock, oxidative stress, and endoplasmic reticulum stress. They are considered essential to protect the tissue from accumulating damage [73]. The essence of this bimodal response to stress is there exists a threshold; the cell mounts a protective response if the stress stimulus does not go above the threshold, while severe stress leads to the activation of stress signalling cascades that fuel into cell death pathways.

### A trimodal response to stress: less adaptive MuSCs spontaneously activate, differentiate, and/or fuse to the myofibre in vivo

**Fig. 4:**
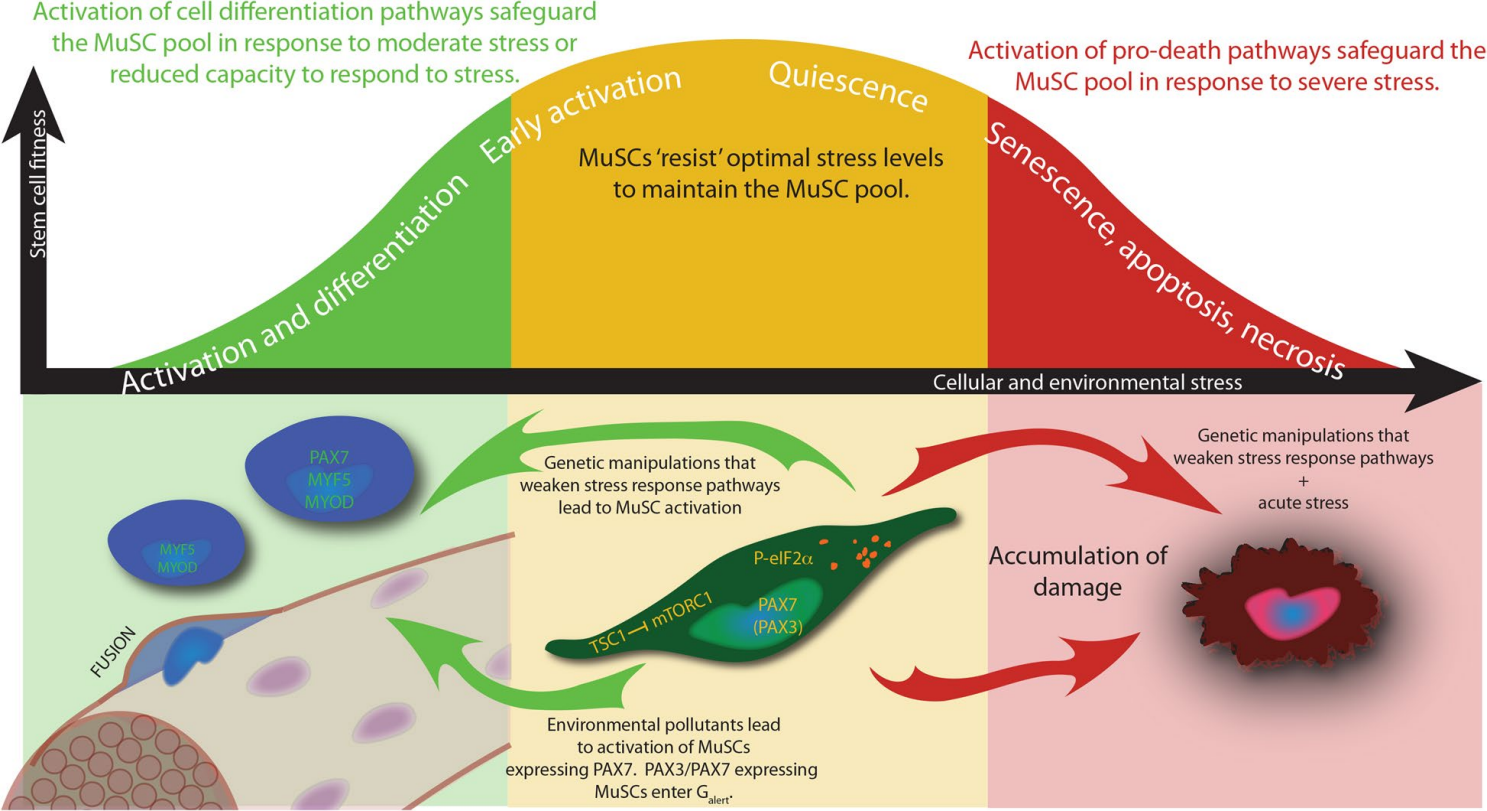
MuSC fitness in relationship to cellular and environmental stress. Cell fates associated with increasing stress are indicated along the modified Yerkes-Dodson curve (white text). (Yellow zone) Optimal levels of cellular and environmental stress are required to maintain the quiescent MuSC pool (dark green cell). Activation of stress response pathways that inhibit protein synthesis, including phosphorylation of eIF2α (P-eIF2α) and TSC1 inhibition of mTORC1 signalling, are required for MuSC quiescence and self-renewal. In addition, a subset of quiescent MuSCs expressing PAX3 (PAX3) exhibit enhanced resistance to stress. Activation of stress response pathways in quiescent MuSCs are also illustrated by the presence of P-eIF2α-dependent RNA granules (orange foci). (Green zone) MuSCs with reduced cellular fitness are removed from the MuSC pool by spontaneous activation (blue cells) and contribution to the myofibre (fusing blue cell with the brown myofibre; myonuclei are indicated in purple). Genetic inactivation of P-eIF2α leads to activation and differentiation of MuSCs. Genetic inactivation of *Tsc1* and exposure to the environmental pollutant TCDD leads to the G_alert_ state of early activation and/or full MuSC activation and differentiation. (Red zone) MuSCs that encounter severe stress, for example accumulating damage associated with aging, or proliferative stress associated with chronic muscle degeneration, are removed from the stem cell pool by the activation of cell senescence or death pathways (bloated red cell)

Within the physiological context of skeletal muscle regeneration, we propose a trimodal response to stress that maintains both the integrity of the MuSC pool and the tissue from accumulating damage (Fig. 4). In addition to pro-cell survival and pro-cell death pathways described above, their exist multiple lines of evidence that MuSCs that have reduced capacity to respond to stress have eliminated themselves from the MuSC pool by spontaneous activation and/or differentiation (Fig. 4). One example is the PERK eIF2α arm of the integrated stress response that is a short-term pro-survival pathway [80, 81]. However, in MuSCs made less resistant to stress by the genetic inactivation of *Perk* or eIF2α phosphorylation, cell death pathways were only activated in response to acute stress induced by thapsigargin treatment in MuSCs cultured ex vivo. Under normal physiological conditions or in response to injury, P-eIF2α defective MuSCs activated and contributed to differentiation, but they did not contribute to the MuSC pool by self-renewal, nor did they undergo apoptosis [7]. A second example is the TSC1/TSC2 inhibitor complex of mTORC1 signalling that is implicated in pro-survival pathways. Mouse embryonic fibroblasts deficient for TSC1/TSC2 exhibit increased levels of apoptosis in response to DNA damage or TNFα stimulation [82]. In contrast, MuSCs made less resistant to stress by the genetic inactivation of *Tsc1* enter the G_alert_ state, the earliest stage of their activation [16, 66]. Third, spontaneous activation and differentiation are also the mechanisms that leads to the depletion of the less stress-resistant, PAX7-only subset of MuSCs that express AhR, when challenged with the environmental stress TCDD [71], while the more stress-resistant *Pax3*-expressing MuSC population enter a G_alert_ state, and may remain present to potentially restore the MuSC pool, as they have been shown to do in response to irradiation [69]. Although these potential activation/differentiation responses to decreased stress resistance are illustrated by genetic perturbations or exposure to environmental stress, it remains unclear whether spontaneous differentiation to protect the stem cell pool is a normal physiological response. Using genetic lineage marker analyses, MuSCs spontaneously activate and fuse to myofibres at unexpected rates [83, 84]. It would be of significant interest to determine whether MuSC activation and fusion to myofibres increase in response to additional stressors below the threshold that would lead to the activation of cell death pathways.

We also highlight that stimulation of stress response pathways enhances MuSC self-renewal and expansion ex vivo. This concept is illustrated by MuSCs that are cultured under low oxygen, which promotes self-renewal and delays differentiation pathways [85]. Pharmacologically maintaining the stress response pathway mediated by P-eIF2α is also effective to expand MuSCs that retain their stem cell regenerative properties ex vivo [7, 60]. Mechanistically, MuSCs may reprogramme translation to P-eIF2α dependent mRNAs, to favour self-renewal or expansion, for example by P-eIF2α-dependent translation of mRNAs like *Tacc3* [61].

## Conclusions

### MuSCs are comfortable being uncomfortable. Cellular and environmental stress tests maintain the MuSC pool

MuSCs activate multiple stress response pathways during quiescence and activation, which seems counterintuitive to the lifelong requirement for these cells to fuel muscle regeneration. These differences may be reconciled if we repurpose the Yerkes-Dodson law, which states that there is an empirical relationship between stress and performance, to stem cell biology (Fig. 4). Quiescent MuSCs might be considered not as a population of cells that exist in comfort to protect them from stress, but rather as a specialised cell population that is continuously challenged with stress as a selective pressure to maintain their fitness, or ‘ability to serve’ in a regenerative response (Fig. 4). Unfit MuSCs may escape stress by spontaneous activation of the myogenic programme and differentiation, if damage is not severe [7, 16, 66, 71] (Fig. 4). Alternatively, if MuSCs encounter severe stress for example in aging and geriatric individuals, or within the context of proliferative stress, they are removed by activation of cell death pathways and/or irreversible senescence [19, 32] (Fig. 4).

A ‘stress test’ is a process to confirm the integrity of critical process by pushing a system to failure. Entities that fail stress tests are removed, while those that pass stress tests are selected to safeguard the system. We propose a cellular stress test for stem cell fitness, whereby quiescent and self-renewing MuSCs pass stress tests administered by their unique prolonged quiescence and microenvironment, which serve as a selective pressure to maintain MuSC fitness and safeguard the lifelong regeneration of the skeletal muscle.

## Data Availability

Not applicable.

## Abbreviations

MuSCs: Muscle stem cells
G_0_: G zero phase, quiescence
*Pax*: Paired box
*Myf5*: Myogenic factor 5
*MyoD*: Myogenic determination protein
RNA: Ribonucleic acid
mRNA: Messenger RNA
eIF2: Eukaryotic inititation factor 2
eIF2α: Eukaryotic intiation factor 2α
P-eIF2α: Phosphorylated eIF2α
ISR: Integrated stress response
p38MAPK: p38-mitogen-activated protein kinase
G_1_: Growth one phase
ATP: Adenosine triphosphate
DMD: Duchenne muscular dystrophy
DTA: Diptheria toxin
*Dmd*^*mdx*^: X-chromosome linked muscular dystrophy mouse model of DMD
eIF4E: Eukaryotic initiation factor 4E
mTORC1: Mechanistic target of rapamycin complex 1
GCN2: General control nonderepressible 2
PKR: Protein kinase R
HRI: Heme-regulated inhibitor
PERK: PKR-like endoplasmic reticulum kinase
met-tRNA: Methionine loaded tRNA
uORF: Upstream open reading frame
5’UTR: 5’ Untranslated region
*Atf4*: Activating transcription factor 4
*Tacc3*: Transforming acidic coiled coil protein 3
PIKK: Phosphatidylinositol 3-kinase (PI3K) – related kinase
4E-BP: eIF4E binding protein 1/2/3 family
p70^S6K^: p70 S6 kinase
m7GTP: 7-Methylguanosine 5-triphosphate
TOP: 5’ Terminal oligopyrmidine
TSC: Tuberous sclerosis
ROS: Reactive oxygen species
DNA: Deoxyribonucleic acid
HGF: Hepatocyte growth factor
DSB: Double-stranded break
DDR: DNA damage reponse
γH2AX: Gamma histone family member X
Ahr: Aryl hydrocarbon receptor
TCDD: 2,3,7,8-Tetrachlorodibenzo-p-dioxin
HSF: Heat shock factor
HSP: Heat shock protein
